# Diversification of non-visual photopigment parapinopsin in spectral sensitivity for diverse pineal functions

**DOI:** 10.1186/s12915-015-0174-9

**Published:** 2015-09-15

**Authors:** Mitsumasa Koyanagi, Seiji Wada, Emi Kawano-Yamashita, Yuichiro Hara, Shigehiro Kuraku, Shigeaki Kosaka, Koichi Kawakami, Satoshi Tamotsu, Hisao Tsukamoto, Yoshinori Shichida, Akihisa Terakita

**Affiliations:** Department of Biology and Geosciences, Graduate School of Science, Osaka City University, Osaka, 558-8585 Japan; Japan Science and Technology Agency (JST), Precursory Research for Embryonic Science and Technology (PRESTO), Kawaguchi, Saitama 332-0012 Japan; Graduate School of Humanities and Science, Nara Women’s University, Nara, 630-8506 Japan; Phyloinformatics Unit, RIKEN Center for Life Science Technologies, Kobe, 650-0047 Japan; Division of Molecular and Developmental Biology, National Institute of Genetics, and Department of Genetics, Sokendai (The Graduate University for Advanced Studies), Mishima, Shizuoka 411-8540 Japan; Department of Biophysics, Graduate School of Science, Kyoto University, Kyoto, 606-8502 Japan

**Keywords:** Animal photoreception, Color vision, Gene duplication, Rhodopsin, Spectral tuning, UV-sensitive pigment

## Abstract

**Background:**

Recent genome projects of various animals have uncovered an unexpectedly large number of opsin genes, which encode protein moieties of photoreceptor molecules, in most animals. In visual systems, the biological meanings of this diversification are clear; multiple types of visual opsins with different spectral sensitivities are responsible for color vision. However, the significance of the diversification of non-visual opsins remains uncertain, in spite of the importance of understanding the molecular mechanism and evolution of varied non-visual photoreceptions.

**Results:**

Here, we investigated the diversification of the pineal photopigment parapinopsin, which serves as the UV-sensitive photopigment for the pineal wavelength discrimination in the lamprey, linking it with other pineal photoreception. Spectroscopic analyses of the recombinant pigments of the two teleost parapinopsins PP1 and PP2 revealed that PP1 is a UV-sensitive pigment, similar to lamprey parapinopsin, but PP2 is a blue-sensitive pigment, with an absorption maximum at 460–480 nm, showing the diversification of non-visual pigment with respect to spectral sensitivity. We also found that PP1 and PP2 exhibit mutually exclusive expressions in the pineal organs of three teleost species. By using transgenic zebrafish in which these parapinopsin-expressing cells are labeled, we found that PP1-expressing cells basically possess neuronal processes, which is consistent with their involvement in wavelength discrimination. Interestingly, however, PP2-expressing cells rarely possess neuronal processes, raising the possibility that PP2 could be involved in non-neural responses rather than neural responses. Furthermore, we found that PP2-expressing cells contain serotonin and aanat2, the key enzyme involved in melatonin synthesis from serotonin, whereas PP1-expressing cells do not contain either, suggesting that blue-sensitive PP2 is instead involved in light-regulation of melatonin secretion.

**Conclusions:**

In this paper, we have clearly shown the different molecular properties of duplicated non-visual opsins by demonstrating the diversification of parapinopsin with respect to spectral sensitivity. Moreover, we have shown a plausible link between the diversification and its physiological impact by discovering a strong candidate for the underlying pigment in light-regulated melatonin secretion in zebrafish; the diversification could generate a new contribution of parapinopsin to pineal photoreception. Current findings could also provide an opportunity to understand the “color” preference of non-visual photoreception.

**Electronic supplementary material:**

The online version of this article (doi:10.1186/s12915-015-0174-9) contains supplementary material, which is available to authorized users.

## Background

Recent genome-wide sequences of various animals have uncovered unexpected diversifications of opsin genes, which encode protein moieties of photoreceptor proteins, in most animals. The biological meaning of the diversification of visual opsins has been well studied; multiple visual opsins with different spectral sensitivities are responsible for color vision and other visual functions [1–3]. Most animals also possess multiple opsins that underlie non-visual functions such as circadian photoentrainment, suggesting their possible diverse and complicated involvement in varied types of non-visual photoreception [4–7]. However, the significance of the diversification of non-visual opsins remains uncertain, although comparative studies of duplicated non-visual opsins such as melanopsin and vertebrate ancient-long (VAL)-opsin have revealed differences in their expression patterns [8–12]. The pineal and related organs of non-mammalian vertebrates are a good model for examining this issue because they are photosensitive organs with diverse functions. One such function is light-dependent regulation of melatonin secretion, which is involved in various physiological functions such as regulation of locomotor activity and sleep [13, 14]. In addition, two types of neural sensing, achromatic responses for luminescence detection and chromatic responses for wavelength discrimination, are well known [15].

Some opsins that serve as so-called pineal photopigments have previously been identified. Pinopsin was the first pineal opsin to be identified in vertebrates, and was originally isolated from chicken pineal organs [16–19]. It has been suggested that pinopsin is involved in light-dependent regulation of melatonin secretion in chicken [16, 20, 21]. Since this discovery, parapinopsin has been identified in the teleost and lamprey pineal and parapineal organs [22, 23], and exo-rhodopsin in the teleost pineal organs [24]. In lizard parietal eyes (a pineal-related organ), the expression of parietopsin as well as pinopsin or parapinopsin has been revealed [25, 26]. The genome-wide sequencing of zebrafish revealed the existence of two genomic DNAs encoding opsins that are highly similar to parapinopsins, indicating that zebrafish have another parapinopsin in addition to the ortholog of parapinopsin originally identified in catfish and rainbow trout [22, 23].

Recently, we used spectroscopic, immunohistochemical and electrophysiological analyses to demonstrate that parapinopsin is the ultraviolet (UV)-sensitive photopigment underlying pineal UV reception in the lamprey [23]. The pineal organs of lampreys and teleosts, as well as the pineal-related organs of frogs (the frontal organ) and lizards (parietal eye), detect the ratio of UV to visible light in the environment [27–33], suggesting that these non-mammalian vertebrates can discriminate between different wavelengths of light using the pineal and related organs, independently of image-forming color vision in the eyes [27]. In previous studies, the electrophysiological response most sensitive to UV light was only observed in chromatic responses for wavelength discrimination and not in achromatic responses for luminescence detection in lamprey and teleost pineal organs [28–30]. We isolated parapinopsin from rainbow trout and clawed frogs, in which the pineal and related organs have been reported to exhibit antagonistic chromatic responses to UV and visible light [29, 32]. In addition, parapinopsin expression in the pineal organ of a closely related species of rainbow trout was recently reported [34]. These facts suggest that parapinopsin may be the common molecular basis for pineal UV reception in wavelength discrimination. This idea is supported by our recent finding that parapinopsin is expressed in the photoreceptor cells of the iguana parietal eye, where the ratio of UV to visible light is detected [26].

Here, we investigated and compared two types of parapinopsins in teleosts, PP1 and PP2, in order to clarify the functional meanings of the diversification. Our spectroscopic, immunohistochemical and transgenic analyses of teleost parapinopsins revealed that PP1 is a UV-sensitive pigment, similar to the lamprey parapinopsin involved in wavelength discrimination, while PP2 is a blue-sensitive pigment that could be involved in melatonin secretion rather than wavelength discrimination. Current findings indicate that the diversification of parapinopsin with respect to spectral sensitivity contributes to other pineal function besides wavelength discrimination, which could be a link between the diversification of non-visual pigments and diverse photoreception in animals.

## Results

### Two parapinopsins in teleosts

**Fig. 1:**
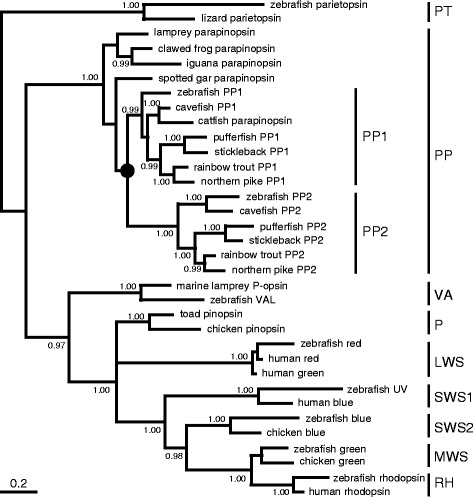
Phylogenetic positions of the two teleost parapinopsins. The molecular phylogenetic tree based on the Bayesian framework contains vertebrate visual and non-visual pigments. Two hundred and fifty six aligned sites were used for the tree inference. Posterior probabilities of more than 0.95 are indicated at branch nodes. The *black circle* indicates the gene duplication that gave rise to two kinds of parapinopsins. LWS long wavelength-sensitive opsin, MWS middle wavelength-sensitive opsin, P pinopsin, PP parapinopsin, PP1 teleost PP1, PP2 teleost PP2, PT parietopsin, RH rhodopsin, SWS1 short wavelength-sensitive type1 opsin, SWS2 short wavelength-sensitive type2 opsin, VA vertebrate ancient opsin. Scale bar = 0.2 substitutions per site

**Fig. 2:**
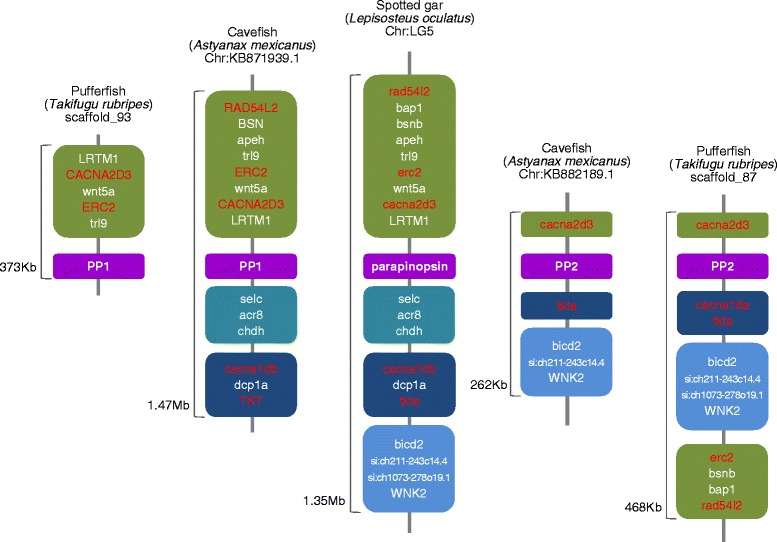
Syntenic regions conserved near to parapinopsin paralogs during teleost evolution. Paralogous genomic regions split by teleost-specific genome duplication are located across the orthologous regions of spotted gar, which split from the teleost lineage prior to the whole genome duplication. *Color boxes* represent the synteny blocks conserved between the paralogous genomic regions or between the teleosts and spotted gar genomes. The genes in *red* indicate the teleost-specific paralogs conserved in the syntenic regions. The detailed synteny map and molecular phylogenetic trees of CACNA2D3/cacna2d3, TKT/tkta, and ERC2/erc2 genes are shown in Additional file 2: Figure S2

Based on two genomic DNA fragments found in the zebrafish genome database that encode opsins which are highly similar (60–70 %) to the lamprey parapinopsin, we isolated two parapinopsin genes (*PP1* and *PP2*) via the homology-based deduction of exons and subsequent full-length cDNA cloning. We also found two parapinopsins in several teleost genome databases, including northern pike, in which pineal antagonistic chromatic responses to UV and visible light were reported [30]. Furthermore, we found another parapinopsin in addition to the previously reported one [23] from the rainbow trout pineal organ, which is also demonstrated to exhibit the antagonistic chromatic responses [29]. These results indicate that teleosts generally have two parapinopsins. The molecular phylogenetic tree of vertebrate visual and non-visual opsins, including these parapinopsins, revealed that teleost PP1s form one cluster with the previously reported catfish parapinopsin, and PP2s form another cluster (Fig. 1, Additional file 1: Figure S1). Interestingly, our comprehensive survey of parapinopsin genes in vertebrate genome databases revealed that the spotted gar has only one parapinopsin, which raises a possibility that the gene duplication giving rise to *PP1* and *PP2* occurred via teleost-specific genome duplication (TSGD) [35, 36] because spotted gar diverged before the TSGD [37, 38]. In fact, the phylogenetic tree based on Bayesian analysis seems to support this scenario, although the statistical support for the tree topology was not strong (Fig. 1). However, the phylogenetic tree inferred with the maximum likelihood method supported another evolutionary relationship, which was also not statistically significant (Additional file 1: Figure S1). We conducted a synteny analysis of genomic regions around the parapinopsin gene(s) in teleosts including the spotted gar to obtain a clue to the timing of the diversification of *PP1* and *PP2*. We found conserved synteny blocks composed of parapinopsin gene(s) and at least five gene families (CACNA2D3/cacna2d3, TKT/tkta, ERC2/erc2, RAD54L2/rad54l2, and cacna1da/cacna1db) between the paralogous genomic regions of some teleosts and between the teleosts and spotted gar genomes (Fig. 2, Additional file 2: Figure S2A). The phylogenetic analyses of three out of the five gene families revealed that the duplications of teleost paralogs occurred after the split of spotted gar, suggesting that the paralogous genomic regions identified in the synteny analysis are derived from TSGD (Additional file 2: Figure S2B-D). Taken together, it is plausible that *PP1* and *PP2* were duplicated via TSGD.

### Diversification of teleost parapinopsin with respect to spectral sensitivity

**Fig. 3:**
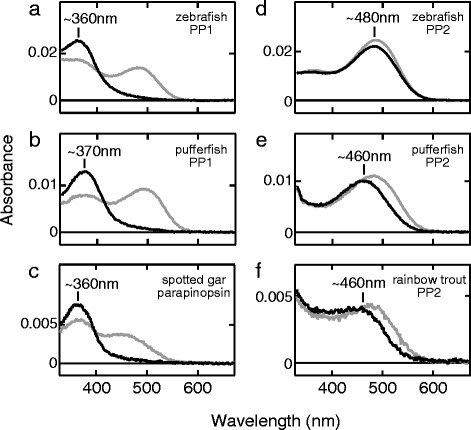
UV-sensitive and blue-sensitive parapinopsins. Absorption spectra in the dark (*black*) and after light-irradiation (*gray*) for **a** zebrafish PP1, **b** pufferfish PP1, **c** spotted gar parapinopsin, **d** zebrafish PP2, **e** pufferfish PP2, and **f** rainbow trout PP2. Note that the absorption spectrum of zebrafish PP2 after light-irradiation (*gray*) was calculated based on the spectral change measured using pre-purified samples (Additional file 3: Figure S3) and the rainbow trout PP1 was not successfully expressed in cultured cells

We investigated and compared the molecular properties of two teleost parapinopsins. We expressed parapinopsins of several teleosts using a mammalian cultured cell expression system, and successfully obtained the purified photosensitive pigments. Spectroscopic analyses showed that the zebrafish and pufferfish PP1s, and spotted gar parapinopsin have absorption maxima at 360–370 nm in the UV region (Fig. 3a–c), indicating that they are UV-sensitive pigments similar to the lamprey parapinopsin [39]. Surprisingly, the zebrafish, pufferfish, and rainbow trout PP2s exhibited absorption maxima in the visible light region, with peaks at ~480 nm, ~460 nm, and ~460 nm, respectively, indicating that teleost PP2 is a blue light-sensitive pigment (Fig. 3d–f). We also found that, regardless of the absorption spectra in the dark, all of these teleost parapinopsins are bistable pigments, which generate a stable photoproduct upon light absorption, and revert to their original state upon subsequent light absorption (Fig. 3; Additional file 3: Figure S3), like the lamprey parapinopsin, [23] and unlike vertebrate visual pigments. These results indicate that parapinopsins have diverse spectral sensitivities (UV and blue), although they are bistable as one of their basic features.

### Mutually exclusive expression of the two parapinopsins in the teleost pineal organ

**Fig. 4:**
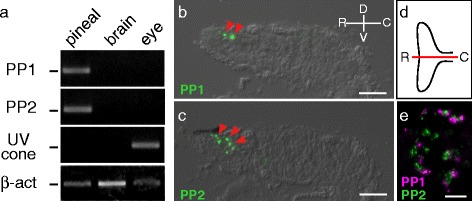
Mutually exclusive localization of PP1 and PP2 in the zebrafish pineal organ. **a** Expression profiles of PP1 (*top panel*), PP2 (*second panel*), UV-sensitive cone opsin (*third panel*) and β-actin as a reference (*bottom panel*) in zebrafish. Expected band sizes of PCR fragments of PP1 (270 bp), PP2 (225 bp), UV-sensitive cone opsin (380 bp), and β-actin (386 bp) based on mobility of standard markers are indicated *on the left*. Immunofluorescent labeling of **b** PP1 and **c** PP2 (*arrowheads*) in the rostral area of the sagittal sections of zebrafish pineal organs. Sections are shown with the dorsal side *up*, ventral side *down*, rostral side *left* and caudal side *right*. **d** Schematic drawing of the dorsal view of the zebrafish pineal organ, rostral side *left* and caudal side *right*. The *red line* indicates the approximate region of the sagittal sections in (b) and (c). **e** Double immunofluorescent labeling of PP1 (*magenta*) and PP2 (*green*) shows the mutually exclusive distribution of PP1 and PP2 in the zebrafish pineal organ. The scale bars represent 20 μm

Next, we investigated the tissue distribution of PP1 and PP2 in zebrafish. Reverse transcription-polymerase chain reaction (RT-PCR) revealed high mRNA expression levels of PP1 and PP2 in the zebrafish pineal organ, but not in the eye or brain (Fig. 4a). This contrasted with the distribution of the UV-sensitive cone opsin (SWS1 opsin), which was expressed in the eye and not in the pineal organ (Fig. 4a). We also conducted immunohistochemical analyses with antibodies specific for the C-terminal region of zebrafish PP1 and PP2 in order to investigate the localization of PP1 and PP2 in the pineal organ in detail. In the zebrafish pineal organ, PP1 and PP2 were localized to the rostral region of the organ, and the distribution of PP2-expressing cells was broader than that of the PP1-expressing cells in the region (Fig. 4b–d). This is in contrast to the broad expression pattern of the major photopigment exo-rhodopsin, which is distributed throughout the zebrafish pineal organ [24]. Immunohistochemical analysis of transverse sections of the rostral pineal organ revealed the mutually exclusive expression of PP1 and PP2 (Fig. 4e). To investigate whether this expression profile is common to teleosts, we also investigated the distribution of PP1 and PP2 in the “I”-shaped pufferfish and rainbow trout pineal organs, which are morphologically different from the “T”-shaped zebrafish pineal organ. In both the pufferfish and rainbow trout pineal organs, PP1 and PP2 were expressed in different photoreceptor cells in the rostral area (Additional file 4: Figure S4, Additional file 5: Figure S5), as observed in the zebrafish pineal organ (Fig. 4). This finding suggests that the expression profile is widely shared among teleosts.

### Different characteristics of PP2-expressing cells from PP1-expressing cells

The finding that PP2 is not a UV-sensitive pigment but a blue-sensitive pigment raised questions about the potential involvement of PP2 in pineal wavelength discrimination. In addition, the mutually exclusive expression of two parapinopsins, which is conserved in three teleost species, allows PP1-expressing and PP2-expressing cells to capture light independently. In order to characterize PP1-expressing and PP2-expressing cells and examine their involvement in pineal wavelength discrimination, we generated transgenic zebrafish in which either PP1-expressing cells or PP2-expressing cells were labeled, because antibodies to opsins essentially label only the outer segments where photopigments are localized, but not the cell bodies or neuronal processes of the photoreceptor cells. We isolated the upstream genomic DNA of zebrafish PP1 (~5.3 kb) and PP2 (~6.7 kb) and introduced green fluorescent protein (GFP) and red fluorescent protein (RFP) into zebrafish under the upstream sequence, respectively. We obtained transgenic zebrafish where GFP and RFP were specifically expressed in the pineal organ (Additional file 6: Figure S6A, B). The GFP and RFP exhibited mutually exclusive expression patterns in the transgenic zebrafish pineal organ (Additional file 6: Figure S6C), which was consistent with the result of the immunohistochemical analysis using antibodies specific for PP1 and PP2 (Fig. 4). The expression patterns of GFP and RFP closely matched those of PP1 and PP2 mRNA revealed by *in situ* hybridization in the transgenic zebrafish pineal organ, respectively (Additional file 7: Figure S7), indicating that these upstream sequences promote specific expression of reporter genes into PP1-expressing and PP2-expresing cells. In the transgenic zebrafish where PP1-expressing cells or PP2-expressing cells were labeled, we detected neuronal processes of PP1-expressing cells, which is consistent with their involvement in pineal wavelength discrimination (Additional file 8: Figure S8A). However, we rarely detected neuronal processes of PP2-expressing cells (Additional file 8: Figure S8B), suggesting the possibility that PP2-expressing cells are mainly involved in non-neural responses rather than neural responses like wavelength discrimination.

### Possible involvement of PP2 in melatonin-secretion

**Fig. 5:**
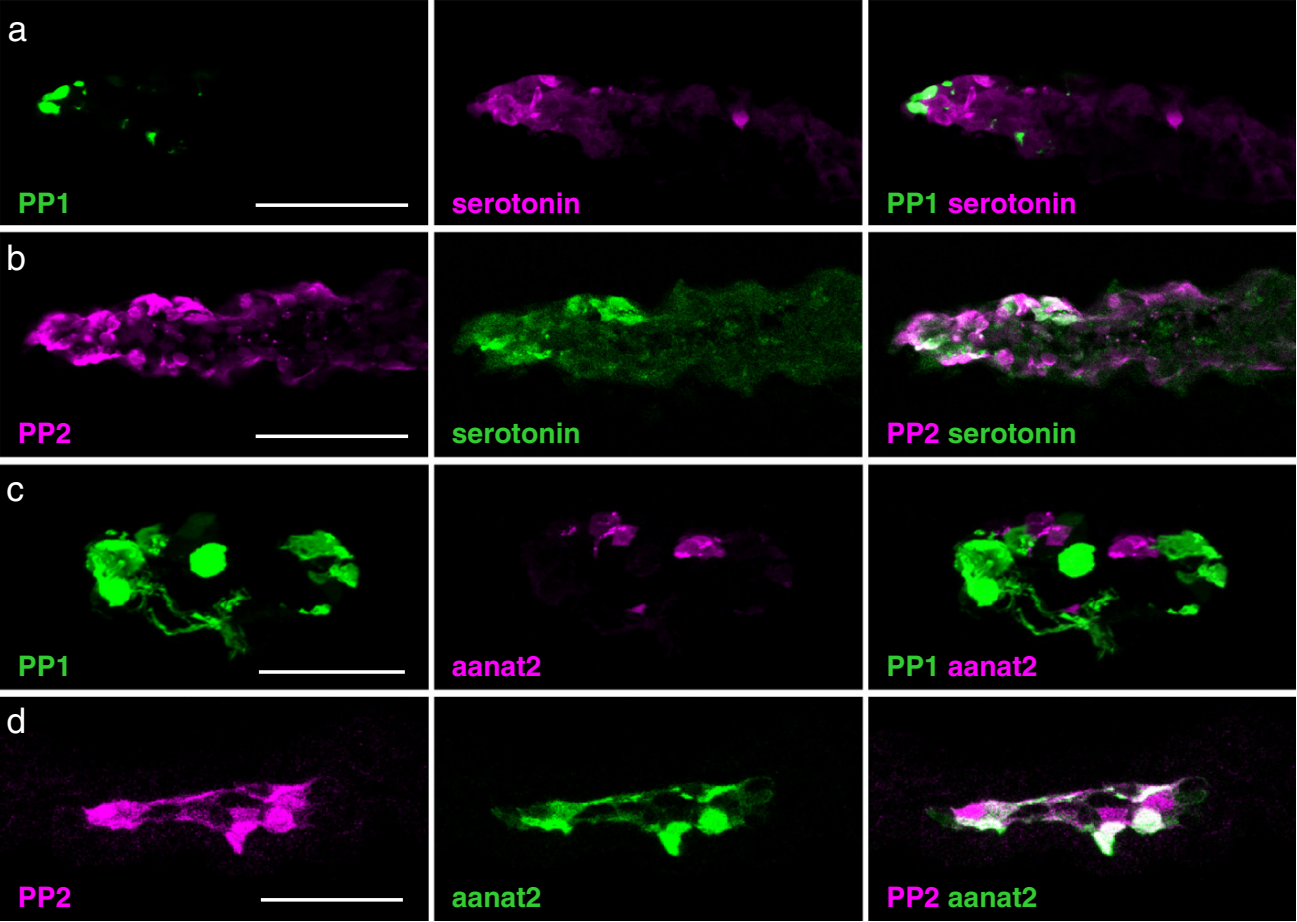
Serotonin and aanat2 localization in PP2-expressing cells analyzed by confocal imaging. Distribution of serotonin-containing cells compared to that of **a** PP1-expressing cells and **b** PP2-expressing cells in the pineal organ of adult zebrafish. RFP and GFP were introduced under the aanat2 promoter into zebrafish where **c** PP1-expressing cells or **d** PP2-expressing cells were labeled with GFP or RFP, respectively. The pineal organs of 6-days-post-fertilization larval zebrafish are shown. The scale bars represent 50 μm (a, b), and 25 μm (c, d)

It is well established that in non-mammalian vertebrates, pineal organs directly capture light to regulate the secretion of melatonin. This so-called pineal hormone is involved in many physiological functions, such as regulation of locomotor activity and sleep [40–44], in addition to neural activities for chromatic and/or achromatic responses. We investigated whether PP2-expressing cells contain serotonin, a precursor of melatonin. Immunohistochemical analysis using the anti-serotonin antibody revealed that RFP-labeled PP2-expressing cells contained large amounts of serotonin, whereas GFP-labeled PP1-expressing cells did not (Fig. 5a, b). We then introduced RFP or GFP under the promoter sequence of arylalkylamine-N-acetyltransferase-2 (aanat2), the key enzyme involved in synthesizing melatonin from serotonin in the pineal organ, into zebrafish where PP1-expressing cells and PP2-expressing cells were labeled with GFP and RFP, respectively. The results showed that aanat2 was expressed in PP2-expressing cells, but not in PP1-expressing cells (Fig. 5c, d). Furthermore, immunohistochemical analysis using antibodies for PP2 and the red-sensitive cone opsin [long wavelength-sensitive (LWS) opsin], which is involved in light-regulated melatonin secretion in various animals [21, 45], revealed that a subset of PP2-expressing cells also expressed LWS opsin in the zebrafish pineal organ (Additional file 9: Figure S9A–C). In light of the finding that PP2 is not a UV-sensitive pigment, these results suggest that in the pineal organ, PP2 is involved in light-regulated melatonin secretion with LWS opsin, rather than wavelength discrimination involving UV reception.

### Spectral tuning mechanism of teleost parapinopsins

**Fig. 6:**
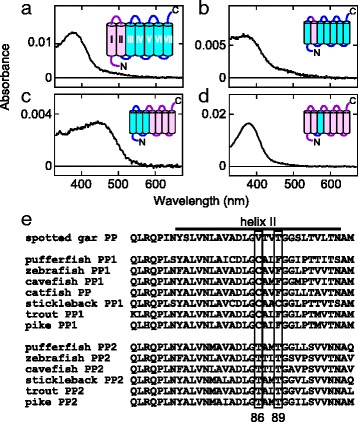
Analyses of chimeric mutants of pufferfish parapinopsin, indicating the importance of helix II in the spectral tuning of teleost parapinopsin. **a–d** Absorption spectra of chimeric mutants (*black curves*) composed of pufferfish PP1 and PP2 helices (*magenta* and *cyan*, respectively, insets). Mutants containing helices I and II of PP1 and helices III–VII of PP2 (a), and the mutant containing only helix II of PP1 in the background of PP2 (b) exhibit their absorption maximum in the UV region. The mutant containing helices I–III of PP2 and helices IV–VII of PP1 (c) exhibits absorption in the visible region, whereas the mutant containing only helix III of PP2 and helices I, II, IV–VII of PP1 (d) remains a UV-sensitive pigment. **e** Comparison of the amino acid sequences of helix II among teleost parapinopsins. The *horizontal bar* on the alignment indicates helix II. Sites 86 and 89 in the parapinopsins are highlighted. Note that mutants containing only helix I of PP1 in the background of PP2 or helices I and/or II of PP2 in the background of PP1 were not expressed

It is interesting to investigate the molecular evolution that resulted in non-visual photopigments with distinct spectral sensitivities via TSGD. We analyzed chimeric mutants of pufferfish PP1 and PP2 because of their high expression level compared to that of other teleost parapinopsin chimeras, in order to obtain a clue to the spectral tuning mechanism of PP1 and PP2. Spectroscopic analyses of a series of chimeric mutants with respect to the transmembrane helix between PP1 and PP2 demonstrated that helix II of PP1 is fully responsible for UV sensitivity, and that helices I–III of PP2 are sufficient to make parapinopsin sensitive to visible light (Fig. 6a–c). In light of the result that introducing helix III of PP2 alone into PP1 did not alter the absorption spectrum (Fig. 6d), these data suggest an important role for helix II in the spectral tuning of teleost parapinopsin, although the mutant containing only helix II of PP2 in the background of PP1 was not expressed. Accumulated mutational analyses revealed that the main site 86 (helix II), as well as other sites in helices I to III (excluding site 89), play crucial roles in the spectral tuning of UV-sensitive cone opsins (SWS1 opsin) [46, 47]. On the other hand, in fruit fly UV-sensitive visual opsin (Rh3), the site equivalent to site 89 in helix II of bovine rhodopsin, site 90, determines the spectral shift between UV-sensitive and blue-sensitive opsins [48]. Therefore, we investigated the contributions of site 86, which is occupied by Val, Cys, and Thr in the gar parapinopsin, PP1s and PP2s, respectively, and site 89, which is occupied by Thr, Phe or Cys and Thr in the gar parapinopsin, PP1s and PP2s, respectively (Fig. 6e). We found no significant spectral changes in teleost parapinopsins in these mutants compared to wild type (Additional file 10: Figure S10). These results suggest that the spectral tuning mechanism of teleost parapinopsins is different from those used by vertebrate SWS1 opsins and fruit fly UV-sensitive visual opsins, even though the importance of helix II is similar in all three of these UV pigments (Fig. 6, Additional file 10: Figure S10).

## Discussion

In this study, we identified two types of parapinopsins, PP1 and PP2, in three kinds of teleosts, and characterized their functions. We found clear differences between PP1 and PP2; PP1 is a UV-sensitive pigment, like the lamprey and iguana parapinopsins [23, 26], but unexpectedly, PP2 is not UV-sensitive and is a novel blue-sensitive parapinopsin (Fig. 3), which shows the diversification of non-visual opsins with respect to spectral sensitivity. Our phylogenetic and synteny analyses suggested that PP1 and PP2 are derived from TSGD [37, 38]. Furthermore, we have clearly shown that the parapinopsin of spotted gar, which diverged before TSGD [37, 38], is a UV-sensitive pigment (Fig. 3c). In addition, based on the fact that the parapinopsins of other vertebrates are also UV-sensitive pigments, we can infer that the blue-sensitive parapinopsin evolved from a UV-sensitive parapinopsin. It is of interest to discuss how the functional differentiations between PP1 and PP2 occurred. In the phylogenetic tree, the branch length between the gene duplication and the deepest node in teleost PP2 (0.291 amino acid substitutions per site) is more than three-fold longer than that between the gene duplication and the deepest node in teleost PP1 (0.086), suggesting a higher accumulation rate of amino acid substitutions in the course of PP2 evolution. On the other hand, sequence comparison of transmembrane helices, which are involved in spectral tuning, between the UV-sensitive parapinopsin group, including the spotted gar parapinopsin, teleost PP1s and other vertebrate parapinopsins, and the teleost PP2 group did not reveal any positions occupied by group-specific amino acid residues with different physicochemical properties other than site 86 (Fig. 6e). Judging from the observation that no significant spectral changes occurred owing to substitutions of amino acid residue at site 86 in pufferfish and zebrafish parapinopsins, multiple amino acid residues, which might be species specific, could be involved in the spectral tuning of parapinopsins. Further mutational analyses including other teleost parapinopsins could reveal a novel spectral tuning mechanism between UV-sensitive and visible-light-sensitive pigments.

We also found that PP1 and PP2 were expressed in different pineal cells in three teleost species belonging to phylogenetically distant teleost orders (Fig. 4, Additional file 4: Figure S4, Additional file 5: Figure S5). These results strongly suggest that the differences in both the molecular properties and expression profiles between PP1 and PP2 are common in teleosts. Furthermore, our characterization of PP1-expressing and PP2-expressing cells in the zebrafish pineal organs revealed that PP1 can be considered a UV-sensitive pigment involved in pineal wavelength discrimination, but, conversely, PP2 is likely involved in light-regulated melatonin secretion (Fig. 5).

We emphasize that the teleost PP1 is a UV-sensitive photopigment (Fig. 3) and its pineal expression is conserved in three kinds of teleosts, including rainbow trout, which has the pineal wavelength discrimination ability [29] (Fig. 4, Additional file 4: Figure S4, Additional file 5: Figure S5). Accordingly, it is strongly suggested that PP1 underlies UV reception for pineal wavelength discrimination in teleosts. Taken together with the involvement of parapinopsins in UV reception in the lamprey pineal organ and iguana parietal eye [23, 26], current findings strengthen the idea that UV-sensitive parapinopsin is a fundamental molecule for wavelength discrimination involving UV reception in the pineal and related organs of non-mammalian vertebrates.

Light-regulated melatonin secretion is the primary function of pineal organs and has therefore been well investigated thus far. However, the underlying photopigments has been largely uncertain in teleosts. We have demonstrated that zebrafish PP2-expressing pineal cells contain serotonin and aanat2 (Fig. 5) and some of them also express LWS opsin, which has been shown to be involved in light-regulated melatonin secretion in the lamprey and chicken [21, 45] (Additional file 9: Figure S9). These results suggest that PP2 and LWS opsin are strong candidates for the light sensor for regulation of melatonin secretion in teleosts. In fact, the combination of PP2 and LWS opsin is consistent with a previous observation, showing that a broad visible light ranging from 400 to 600 nm influences melatonin secretion in zebrafish pineal organs [49]. This is similar to the case of the chicken pineal organ, where the blue-sensitive pigment pinopsin and LWS opsin are suggested to be involved in melatonin secretion [16, 20, 21]. Interestingly, teleosts with PP2 do not possess pinopsin in their genomes whereas the spotted gar, which does not contain PP2, has pinopsin. These observations lead us to speculate that blue light might be suitable for the regulation of melatonin secretion, with the evolution of PP2 potentially being related to the loss of pinopsin in teleosts. Accordingly, our findings provide a clear-cut example for functional differentiation among paralogs; the teleost PP1 could have retained the putative ancestral function, whereas teleost PP2 acquired a novel functional role via a neofunctionalization event.

## Conclusions

Recent genome projects of many animals have revealed that non-visual opsins have undergone a number of diversifications. However, the functional meaning of these diversifications remain unclear, in contrast to those of visual opsins, which diversified in spectral sensitivity to enable color vision and other visual function [1–3]. In this paper, we have clearly shown the differences in the molecular properties of duplicated non-visual opsins, by demonstrating the diversification of parapinopsin with respect to spectral sensitivity, which generated a blue-sensitive parapinopsin from the original UV-sensitive parapinopsin. Moreover, we also showed a plausible link between this diversification and its physiological consequences; this diversification could allow parapinopsin to contribute to the light regulation of melatonin secretion, in addition to wavelength discrimination. Current findings that two parapinopsins with different spectral sensitivities could contribute to different pineal functions (wavelength discrimination and melatonin secretion) also provide an opportunity to improve our understanding of the “color” preferences of non-visual photoreception.

## Methods

### Ethics statement

This experiment was approved by the Osaka City University animal experiment committee (#S0032, #514) and complied with the Regulations on Animal Experiments from Osaka City University.

### Animals

Zebrafish (*Danio rerio*) were obtained from the Zebrafish International Resource Center (ZIRC) and National Bio Resource Project (NBRP) Zebrafish. Pufferfish (*Takifugu rubripes*), rainbow trout (*Oncorhynchus mykiss*), and spotted gar (*Lepisosteus oculatus*) were commercially obtained.

### cDNA cloning

Partial cDNAs of parapinopsins of the pufferfish, zebrafish, and spotted gar were isolated from the RNA of pineal organs by RT-PCR using gene-specific primers based on gene sequences found in genome databases. A partial cDNA of the rainbow trout PP2 was isolated from the RNA of pineal organs by RT-PCR using the following degenerate primers: 5′-TGYACiGTiGCiYTNATHGCNGT-3′ as the sense primer and 5′-GAATTCAIIGCRTADATIAINGGRTT-3′ as the antisense primer. These primers were based on CTVALIAV and NPIIYAL, respectively. PCR amplifications using degenerate primers were carried out at an annealing temperature of 40 °C as described [50]. Full-length cDNAs of the parapinopsins were obtained using the 3′ RACE and 5′ RACE systems (Invitrogen, Life Technologies, Carlsbad, California, USA).

### Phylogenetic analyses

Amino acid sequences of vertebrate visual and non-visual pigments, including parapinopsins, were aligned with MAFFT version 7.221 employing the L-INS-i method [51]. Unambiguously aligned sites were selected with trimAl version 1.4 using the automated1 option [52] followed by trimming gapped sites. A molecular phylogenetic tree was inferred with PhyloBayes 4.1b, which employs the Bayesian framework, assuming the CAT-GTR model [53]. The PhyloBayes run performed 10,900 cycles of MCMC chain from which the first 1,000 cycles were discarded as burn-in. A phylogenetic tree based on the maximum-likelihood approach was inferred with RAxML version 8.1.22 assuming the PROTCATGTR model [54]. One thousand bootstrap replicates were performed for maximum-likelihood tree inference (“-f a” option for RAxML). The accession numbers of the sequences used for the phylogenetic tree inference are provided in Additional file 11.

### Synteny analysis

Syntenic regions near parapinopsin genes in bony fish genomes were identified by the Genomicus database version 80.01 [55]. Three gene families, CACNA2D3/cacna2d3, TKT/tkta, and ERC2/erc2, in which the paralogs generated in teleost-specific genome duplication were both conserved in at least one teleost species, were subject to phylogenetic analysis. Amino acid sequences of these genes were obtained with the aid of a web tool, aLeaves [56]. Bayesian phylogenetic trees of these genes were inferred with the procedures described above. Ambiguous sites of the ERC2/erc2 amino acid sequence alignment were removed by visual inspection as well as with trimAl. The PhyloBayes runs were performed for 13,300, 19,800, and 4,200 cycles of MCMC chain for CACNA2D3/cacna2d3, TKT/tkta, and ERC2/erc2 genes, respectively, from which the first 1,000 cycles were discarded as burn-in.

### Expression of the opsin-based pigment and spectroscopy

The cDNAs of the parapinopsins and their various mutants were tagged with the monoclonal antibody Rho 1D4 epitope sequence (ETSQVAPA). The tagged cDNA was inserted into a pcDNA3.1 plasmid vector (Invitrogen). Chimeric mutants with respect to the transmembrane helix between PP1 and PP2 were generated by combining two cDNA fragments using PCR with primers at the ends of the combined sequence. R55/Q56, M89/G90, N130/P131, D168/L169, A226/E227, and Y270/I271 in the pufferfish PP1, and K56/Q57, Q90/G91, K131/P132, E169/L170, M227/E228, and K271/I272 in the pufferfish PP2 are boundaries of helices I/II, II/III, III/IV, IV/V, V/VI, and VI/VII, respectively. A point mutation was introduced into the cDNA with a commercial site-directed mutagenesis kit, QuikChange (Stratagene, Agilent Technologies, Santa Clara, California, USA), according to the manufacturer’s instructions. Pigment expression in HEK293S cells and purification were carried out as previously described [57]. Briefly, to reconstitute the pigment, the expressed proteins were incubated with excess 11-*cis* retinal overnight. The pigments were then extracted with 1 % dodecyl β-D-maltoside (DM) in 50 mM HEPES buffer (pH 6.5) containing 140 mM NaCl (buffer A). To purify the pigment, pigments in the crude extract were bound to 1D4-agarose, washed with 0.02 % DM in buffer A (buffer B) and eluted with buffer B that contained the 1D4 peptide. The absorption spectra of pigments were recorded at 4 °C with a Shimadzu UV2450 spectrophotometer. UV, blue, and orange light were supplied by a 1-kW halogen lamp (Philips, Eindhoven, Netherlands) with a UV glass filter, UTVAF-50S-36U (Sigma Koki, Saitama, Japan), a 460-nm interference filter, and an O56 glass cutoff filter (Toshiba, Tokyo, Japan), respectively.

### RT-PCR analysis

mRNA from pineal organ, brain, and eye of zebrafish was purified with an Oligotex -dT30 Super mRNA Purification Kit (TaKaRa, Otsu, Japan), and the mRNAs were reverse transcribed to cDNAs with a High Capacity cDNA Reverse Transcription Kit (Applied Biosystems, Waltham, Massachusetts, USA). The β-actin genes were used as internal references. The primer sequences used for amplification of each gene and the length of amplicons are as follows. 5′-TGCGGCAGGTGAGTCGTCTG-3′ and 5′-GATCCCTGAACTGTCTGTTC-3′ for amplification of a 270-bp fragment of PP1, 5′-GCTGAGACAAGTTGCTAAGG-3′ and 5′-ACCTCTGGAACTGTTTGTTC-3′ for amplification of a 225-bp fragment of PP2, 5′-TCGATTGCAGGTCTTGTGAC-3′ and 5′- TGGGTGGACTCTGACTCGGC-3′ for amplification of a 380-bp fragment of SWS1 opsin, and 5′-TGGAGAAGAGCTATGAGCTG-3′ and 5′-ACTCATCGTACTCCTGCTTG-3′ for amplification of a 386-bp fragment of β-actin.

### Antibodies and immunohistochemistry

The anti-zebrafish PP1, anti-zebrafish PP2, anti-pufferfish PP1, and anti-pufferfish PP2 antibodies were generated against the C-terminal 50-amino-acid region of the protein. The anti-rainbow trout PP1 and anti-rainbow trout PP2 antibodies were generated against the C-terminal 40 amino acids and 44 amino acids, respectively. The anti-zebrafish LWS opsin was generated against the C-terminal 41-amino-acid region, which is conserved in two zebrafish LWS opsins, LWS1 and LWS2 [58]. The antigens were prepared using the pMAL Protein Fusion and Purification System (New England Biolabs, Ipswich, Massachusetts, USA), according to a previously reported method [59]. The dissected pineal organs with brain were immersion-fixed in 4 % paraformaldehyde, cryoprotected in 0.1 M phosphate buffer containing 30 % sucrose, frozen with OCT Compound (Sakura Finetechnical, Tokyo, Japan), and sectioned at 12–20 μm. The sections were incubated with 1:500 diluted antisera, followed by incubation with Alexa Fluor 488 or 594 anti-mouse IgG or Alexa Fluor 488 or 594 anti-rabbit IgG (Molecular Probes, Life technologies, Carlsbad, California, USA) for immunofluorescent detection. For immunohistochemical labeling of serotonin, the anti-serotonin antibody (ImmunoStar, Hudson, Wisconsin, USA) was used. The anti-GFP antibody (Nacalai Tesque, Kyoto, Japan) and anti-DsRed antibody (Clontech, Mountain View, California, USA) were used to visualize the GFP and RFP, respectively. Confocal images were obtained with confocal laser scanning microscopes C1 siReady (Nikon, Tokyo, Japan) or Leica TCS SP8 (Leica, Wetzlar, Germany).

### Generating and analyzing transgenic zebrafish

The 5,293-bp and 6,719-bp upstream sequence from the ATG initiation codon of zebrafish PP1 and PP2 genes, respectively, were obtained from zebrafish genomic DNA by PCR. The promoter sequence of aanat2 was obtained according to a previous report [60]. Each DNA fragment was inserted into the *Tol2* vector pT2AL200R150G, which contains the GFP expression cassette [61]. To introduce RFP, the GFP cDNA in the vector was replaced by mRFP cDNA obtained from pT2ZUASRFP [62]. Microinjection of the construct DNA and transposase mRNA to zebrafish zygotes was carried out as previously reported [61]. We also generated the transgenic line where Gal4 is introduced under the PP1-promoter and the F1 fish from the Gal4 line and UAS-GFP line [62] was used to examine the presence of serotonin and aanat2 in the PP1-expressing cells. For labeling LWS1-expressing and LWS2-expressing cells, Tg(LWS1up2.6 kb:GFP)#1509 and Tg(LAR:LWS2up1.8 kb:GFP)#1499 were used [63].

### *In situ* hybridization

Digoxigenin (DIG)-labeled antisense and sense RNA probes for zebrafish PP1 and PP2 mRNAs were synthesized using the DIG RNA labeling kit (Roche, Basel, Switzerland). Sections were pretreated with proteinase K and hybridized with each RNA probe in ULTRAhyb Ultrasensitive Hybridisation Buffer (Ambion, Life Technologies, Carlsbad, California, USA). The probe on the sections was detected by incubation with alkaline phosphatase-conjugated anti-DIG antibody (Roche) followed by a blue 5-bromo-4-chloro-3-indolyl phosphate/nitro blue tetrazolium color reaction.

### Data deposition

The sequences reported in this paper have been deposited in the DDBJ database [accession nos. AB626964 - AB626967, AB675727].

## Additional files

Additional file 1: Figure S1. Phylogenetic tree of the two teleost parapinopsins based on maximum-likelihood approach. (PDF 142 kb) Additional file 2: Figure S2. Detailed synteny map and molecular phylogenetic trees of CACNA2D3/cacna2d3, TKT/tkta, and ERC2/erc2 genes. (PDF 188 kb) Additional file 3: Figure S3. Bistable nature of the blue-sensitive parapinopsin PP2. (PDF 181 kb) Additional file 4: Figure S4. Distribution of PP1 and PP2 in the pineal organ of pufferfish. (PDF 6217 kb) Additional file 5: Figure S5. Distribution of PP1 and PP2 in the pineal organ of rainbow trout. (PDF 873 kb) Additional file 6: Figure S6. Pineal-specific and mutually exclusive expression of GFP and RFP introduced under the upstream sequences of the PP1 gene and PP2 gene, respectively, in zebrafish. (PDF 514 kb) Additional file 7: Figure S7. Labeling of PP1-expressing and PP2-expressing cells in the zebrafish pineal organ. (PDF 2686 kb) Additional file 8: Figure S8. Comparison of cell morphology between PP1-expressing and PP2-expressing cells. (PDF 276 kb) Additional file 9: Figure S9. Characterization of LWS opsin-expressing cells in the zebrafish pineal organ. (PDF 3655 kb) Additional file 10: Figure S10. Spectroscopic analyses of teleost parapinopsin mutants. (PDF 147 kb) Additional file 11:
**Accession numbers of amino acid sequences used in the molecular phylogeny inference.** (PDF 30 kb)
