# Supplementary material for: Diversification of non-visual photopigment parapinopsin in spectral sensitivity for diverse pineal functions
Source: BMC Biol. 2015 Sep 15;13:73. doi: 10.1186/s12915-015-0174-9 (PMC4570685; doi:10.1186/s12915-015-0174-9)
Supplement: Additional file 1: Figure S1. — Phylogenetic tree of the two teleost parapinopsins based on maximum-likelihood approach. (PDF 142 kb) [file 12915_2015_174_MOESM1_ESM.pdf]

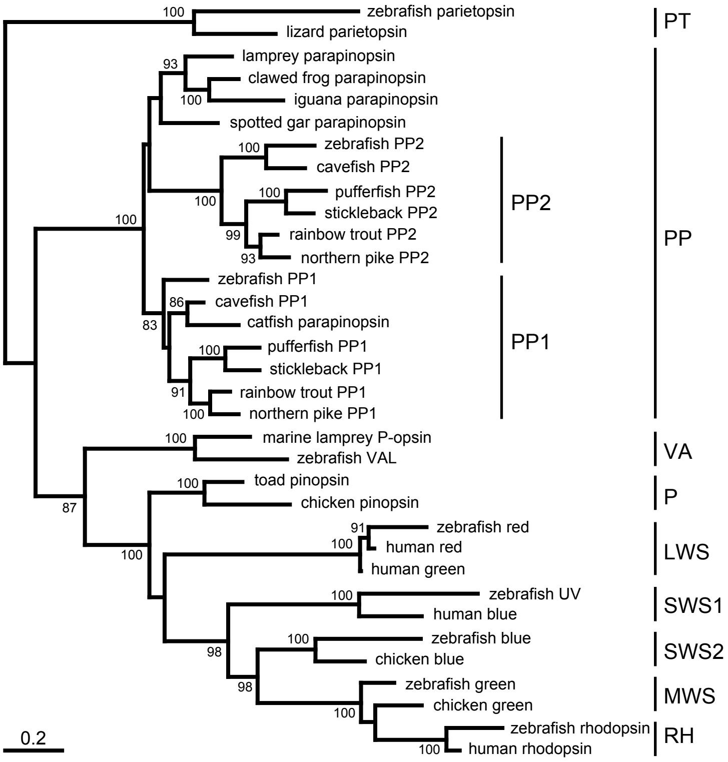

Figure S1. Phylogenetic tree of the two teleost parapinopsins based on maximum-likelihood approach. Bootstrap probabilities of more than 70% are shown at branch nodes. See Figure 1 for the details. Scale bar = 0.2 substitutions per site.
