# Supplementary material for: Diversification of non-visual photopigment parapinopsin in spectral sensitivity for diverse pineal functions
Source: BMC Biol. 2015 Sep 15;13:73. doi: 10.1186/s12915-015-0174-9 (PMC4570685; doi:10.1186/s12915-015-0174-9)
Supplement: Additional file 2: Figure S2. — Detailed synteny map and molecular phylogenetic trees of CACNA2D3/cacna2d3, TKT/tkta, and ERC2/erc2 genes. (PDF 188 kb) [file 12915_2015_174_MOESM2_ESM.pdf]

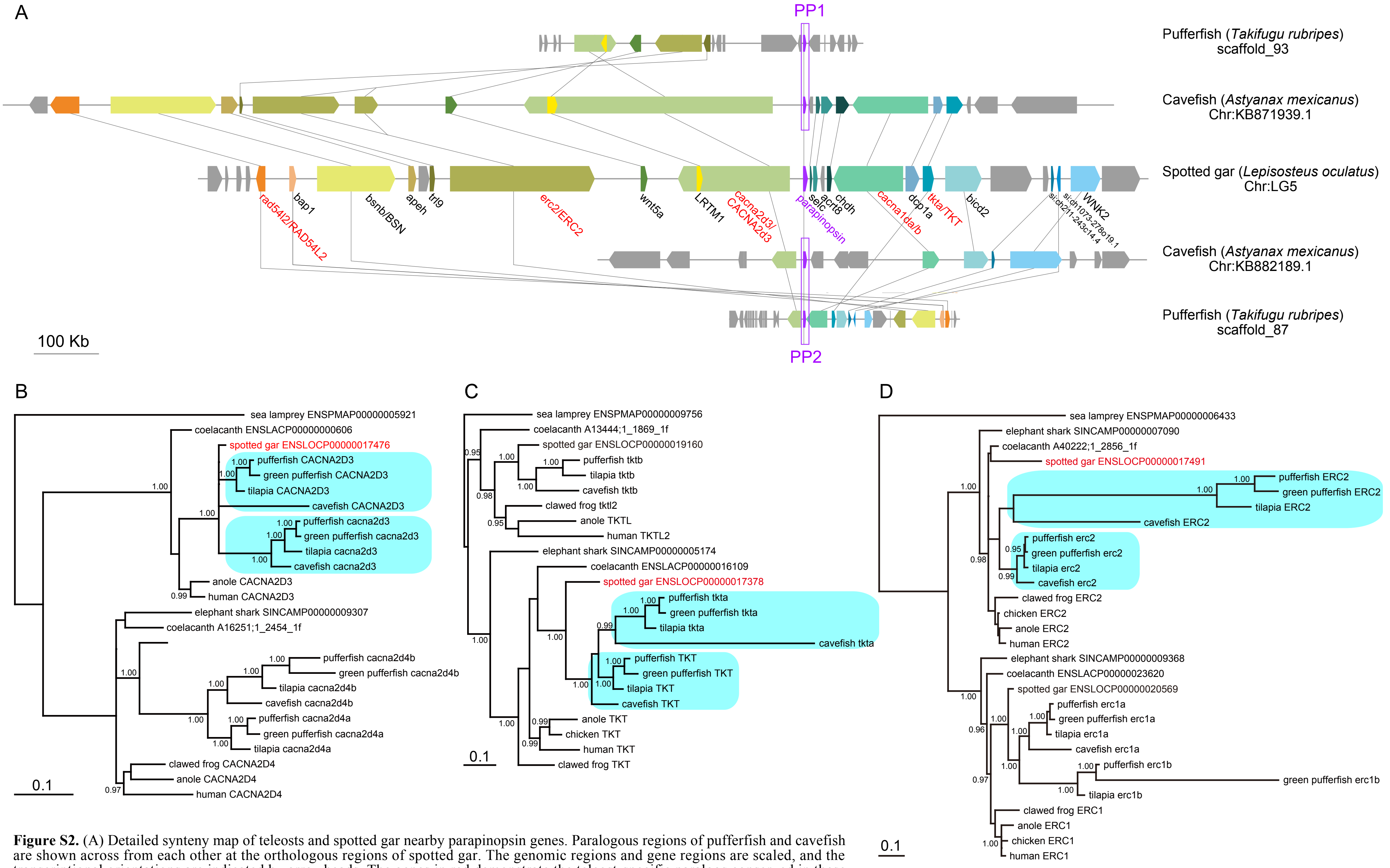

Figure S2. (A) Detailed synteny map of teleosts and spotted gar nearby parapinopsin genes. Paralogous regions of pufferfish and cavefish are shown across from each other at the orthologous regions of spotted gar. The genomic regions and gene regions are scaled, and the transcriptional orientations are indicated by arrowheads. The genes in red demonstrate the teleost-specific paralogs conserved in these syntenic regions. This figure is modified from the synteny map provided by the Genomicus database [55]. The molecular phylogenetic trees of CACNA2D3/cacna2d3 (B), TKT/tkta (C), and ERC2/erc2 (D) genes: both of the teleost-specific paralogs are conserved in the syntenic regions in at least one species. For the tree inference of CACNA2D3/cacna2d3, TKT/tkta, and ERC2/erc2 genes, 408, 422, and 532 aligned sites are used, respectively. In the phylogenetic trees, spotted gar orthologs are shown in red, and the teleost-specific paralogs are shown with light-blue backgrounds. See Fig. 1 for the details in tree view and Additional file 11 for sequence IDs except for those of spotted gar, coelacanth, elephant shark, and sea lamprey, which are indicated in the trees. Scale bar = 0.1 substitutions per site.
