# Supplementary material for: Diversification of non-visual photopigment parapinopsin in spectral sensitivity for diverse pineal functions
Source: BMC Biol. 2015 Sep 15;13:73. doi: 10.1186/s12915-015-0174-9 (PMC4570685; doi:10.1186/s12915-015-0174-9)
Supplement: Additional file 3: Figure S3. — Bistable nature of the blue-sensitive parapinopsin PP2. (PDF 181 kb) [file 12915_2015_174_MOESM3_ESM.pdf]

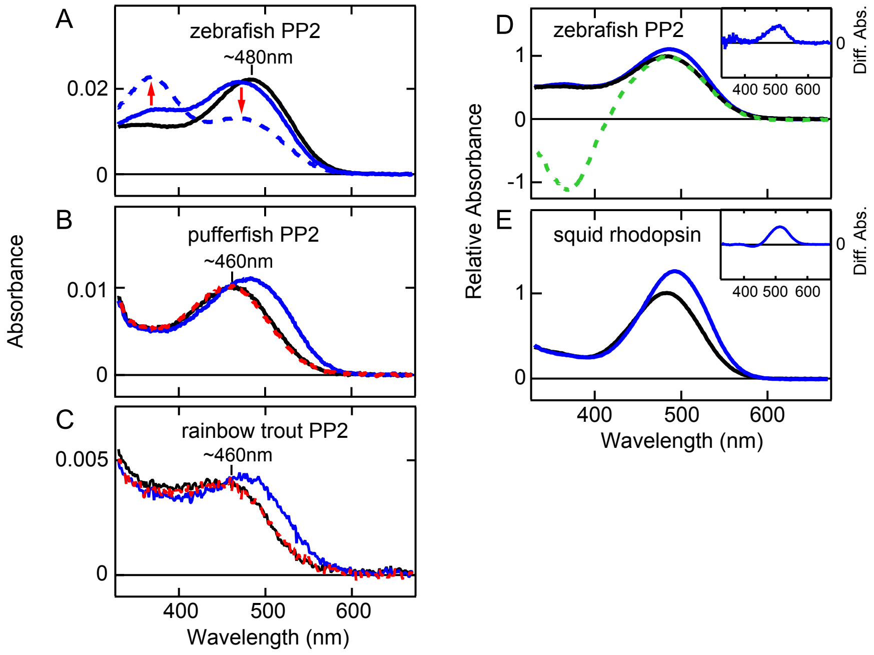

Figure S3. Bistable nature of the blue-sensitive parapinopsin PP2. Absorption spectra of purified zebrafish PP2 (A), pufferfish PP2 (B), and rainbow trout PP2 (C). Blue light-irradiation caused a red-shift of the spectra (blue curves) of pufferfish PP2 (B) and rainbow trout PP2 (C), indicating formation of stable photoproducts with absorption maxima in the visible light region. Subsequent orange light-irradiation restored the pigments to their original dark states (broken red curves). The photoproduct of the purified zebrafish PP2 was unstable and gradually decayed (A). The solid blue curve and the broken blue curve in (A) represent the absorption spectra obtained immediately after and 30 min after blue light-irradiation, respectively. However, the difference spectrum (after minus before irradiation) of the pre-purified zebrafish PP2 under neutral conditions (pH 6.5) (D, inset) was stable and was not changed by orange light-irradiation, in a manner similar to a typical bistable pigment squid rhodopsin (E), showing that zebrafish PP2 is also a bistable pigment. This unchanged spectral property of bistable pigments is explained by the following. A bistable pigment whose dark state and photoproduct have almost identical wavelengths of absorption maxima exhibits almost the same absorption spectra after irradiation with any color of light, as in the case of squid rhodopsin (E). An absolute spectrum of the zebrafish PP2 photoproduct was calculated using pre-purified zebrafish PP2, according to the previously reported method [64](D). The black curve represents the absolute dark spectrum of the zebrafish PP2 that was normalized to fit the difference spectrum (before minus after irradiation) of the pre-purified zebrafish PP2 under alkaline conditions (pH 9.8) (broken green curve) in the long-wavelength region. The calculated absorption spectrum of the zebrafish PP2 photoproduct (blue curve) was obtained by adding the difference spectrum (after minus before irradiation) of the pre-purified zebrafish PP2 under neutral conditions (pH 6.5) (inset) to the normalized dark spectrum of zebrafish PP2 (black curve). These procedures revealed that the zebrafish PP2 photoreaction involves the generation of a stable photoproduct with an absorption maximum in the visible light region and a larger extinction coefficient. (E) The absolute spectra of squid rhodopsin before (black curve) and after light-irradiation (blue curve) and their difference spectrum (inset).
