# Supplementary material for: Diversification of non-visual photopigment parapinopsin in spectral sensitivity for diverse pineal functions
Source: BMC Biol. 2015 Sep 15;13:73. doi: 10.1186/s12915-015-0174-9 (PMC4570685; doi:10.1186/s12915-015-0174-9)
Supplement: Additional file 4: Figure S4. — Distribution of PP1 and PP2 in the pineal organ of pufferfish. (PDF 6217 kb) [file 12915_2015_174_MOESM4_ESM.pdf]

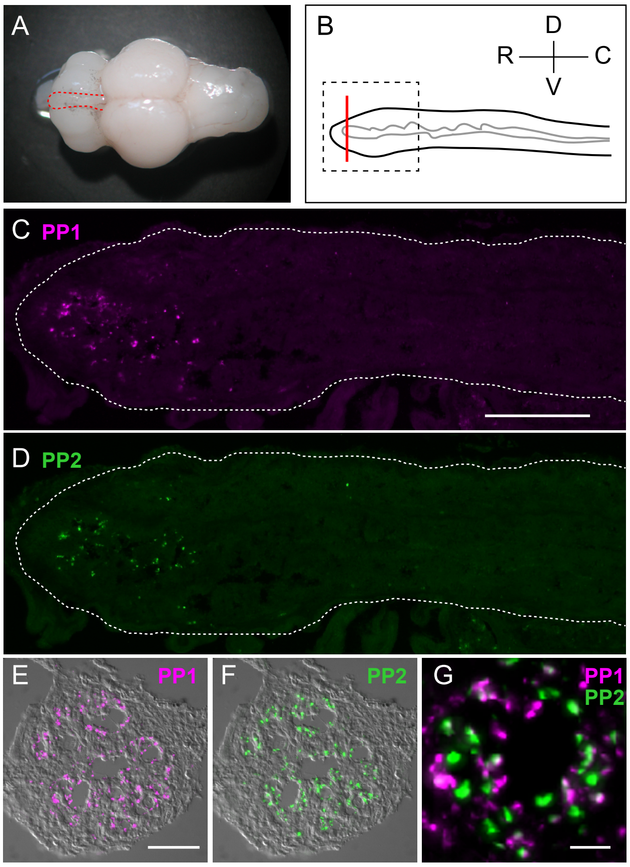


**Figure S4.** Distribution of PP1 and PP2 in the pineal organ of pufferfish. (A) Dorsal view of the pufferfish brain. The dotted trace indicates the landmark of the pineal organ. (B) Schematic drawing of the pufferfish pineal organ, dorsal side up, ventral side down, rostral side left, and caudal side right. The gray line indicates the landmark of the pineal lumen. Immunofluorescent labeling of PP1 (C) and PP2 (D) in the rostral area of the pineal organ. The white dotted traces in (C) and (D) indicate the landmarks of the pineal organ, corresponding to the region indicated by the box in (B). The distributions of PP1 (E) and PP2 (F) in a transverse section of the rostral pineal organ indicated by the red line in (B). (G) High-magnification merged image of (E) and (F), showing the mutually exclusive distribution of PP1 and PP2. The scale bars represent 200 mm in (C), 50 mm in (E) and 10 mm in (G)
