# Supplementary material for: Diversification of non-visual photopigment parapinopsin in spectral sensitivity for diverse pineal functions
Source: BMC Biol. 2015 Sep 15;13:73. doi: 10.1186/s12915-015-0174-9 (PMC4570685; doi:10.1186/s12915-015-0174-9)
Supplement: Additional file 5: Figure S5. — Distribution of PP1 and PP2 in the pineal organ of rainbow trout. (PDF 873 kb) [file 12915_2015_174_MOESM5_ESM.pdf]

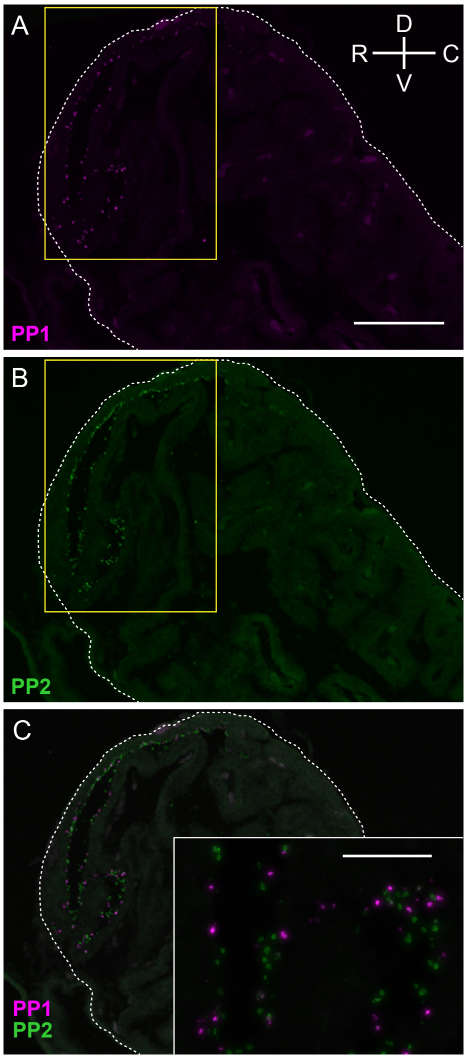

Figure S5. Distribution of PP1 and PP2 in the pineal organ of rainbow trout. Immunohistochemical analysis revealed the localization of PP1 (A) and PP2 (B) in the rostral area (boxed) of the sagittal sections of the rainbow trout pineal organ. (C) Merged image of (A) and (B). (Inset) High-magnification image, clearly showing the mutually exclusive distribution of PP1 and PP2. Sections are shown with the dorsal side up, ventral side down, rostral side left and caudal side right. The scale bars represent 200 μm in (A) and 50 μm in inset of (C).
