# Supplementary material for: Diversification of non-visual photopigment parapinopsin in spectral sensitivity for diverse pineal functions
Source: BMC Biol. 2015 Sep 15;13:73. doi: 10.1186/s12915-015-0174-9 (PMC4570685; doi:10.1186/s12915-015-0174-9)
Supplement: Additional file 6: Figure S6. — Pineal-specific and mutually exclusive expression of GFP and RFP introduced under the upstream sequences of the PP1 gene and PP2 gene, respectively, in zebrafish. (PDF 514 kb) [file 12915_2015_174_MOESM6_ESM.pdf]

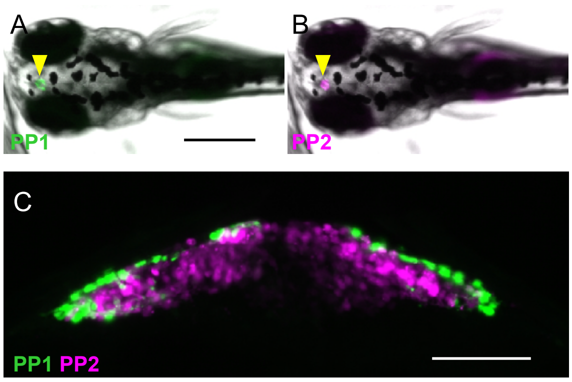

Figure S6. Pineal-specific and mutually exclusive expression of GFP and RFP introduced under the upstream sequences of the PP1 gene and PP2 gene, respectively in zebrafish. GFP and RFP expression (arrowheads) driven by ~5.3 kb upstream sequence of the PP1 gene (A) and ~6.7 kb upstream sequence of PP2 gene (B), respectively in zebrafish larva (7 dpf). (C) In the transverse section of the pineal organ of the transgenic zebrafish (adult), the GFP and RFP exhibited mutually exclusive expression. The scale bars represent 200 µm in (A), and 50 µm in (C).
