# Supplementary material for: Diversification of non-visual photopigment parapinopsin in spectral sensitivity for diverse pineal functions
Source: BMC Biol. 2015 Sep 15;13:73. doi: 10.1186/s12915-015-0174-9 (PMC4570685; doi:10.1186/s12915-015-0174-9)
Supplement: Additional file 7: Figure S7. — Labeling of PP1-expressing and PP2-expressing cells in the zebrafish pineal organ. (PDF 2686 kb) [file 12915_2015_174_MOESM7_ESM.pdf]

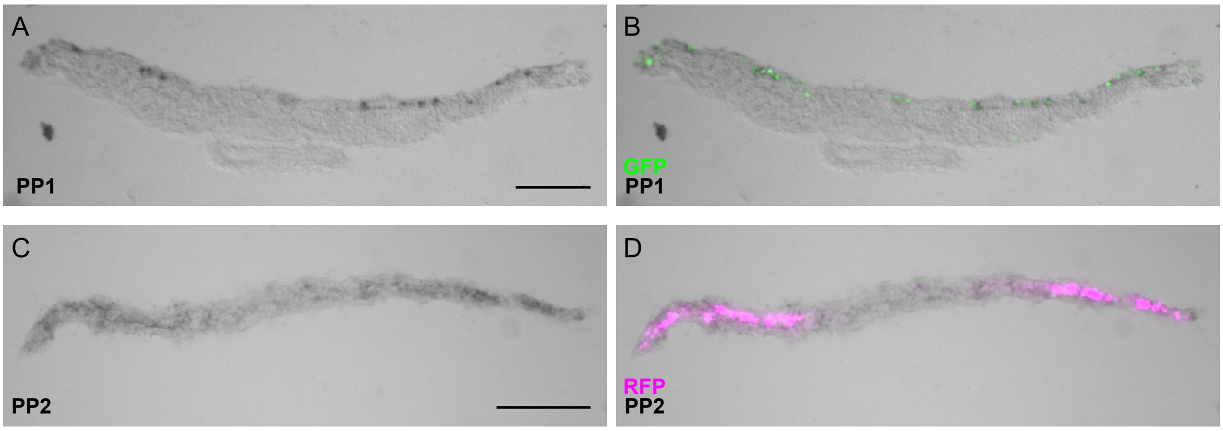

Figure S7. Labeling of PP1-expressing and PP2-expressing cells in the zebrafish pineal organ. Expression patterns of PP1 (A) and PP2 (C) in transverse sections of the rostral area of the adult pineal organ, revealed by *in situ* hybridization matched well to those of GFP (B) and RFP (D), respectively, in transgenic zebrafish. Note that the images of the GFP and RFP signals were captured prior to the *in situ* hybridization procedure, and merged with that of *in situ* hybridization samples based on obvious landmarks in the pineal organ. The scale bars represent 100 μm.
