# Supplementary material for: Diversification of non-visual photopigment parapinopsin in spectral sensitivity for diverse pineal functions
Source: BMC Biol. 2015 Sep 15;13:73. doi: 10.1186/s12915-015-0174-9 (PMC4570685; doi:10.1186/s12915-015-0174-9)
Supplement: Additional file 8: Figure S8. — Comparison of cell morphology between PP1-expressing and PP2-expressing cells. (PDF 276 kb) [file 12915_2015_174_MOESM8_ESM.pdf]

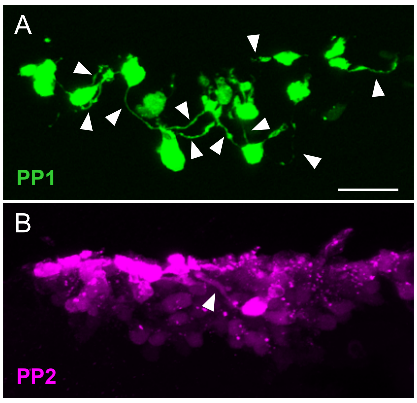

Figure S8. Comparison of cell morphology between PP1-expressing and PP2-expressing cells. Neuronal processes (arrowheads) were observed in many GFP-labeled PP1-expressing cells (A), but rarely in RFP-labeled PP2-expressing cells (B). The scale bar represents 20 μm.
