# Supplementary material for: Diversification of non-visual photopigment parapinopsin in spectral sensitivity for diverse pineal functions
Source: BMC Biol. 2015 Sep 15;13:73. doi: 10.1186/s12915-015-0174-9 (PMC4570685; doi:10.1186/s12915-015-0174-9)
Supplement: Additional file 9: Figure S9. — Characterization of LWS opsin-expressing cells in the zebrafish pineal organ. (PDF 3655 kb) [file 12915_2015_174_MOESM9_ESM.pdf]

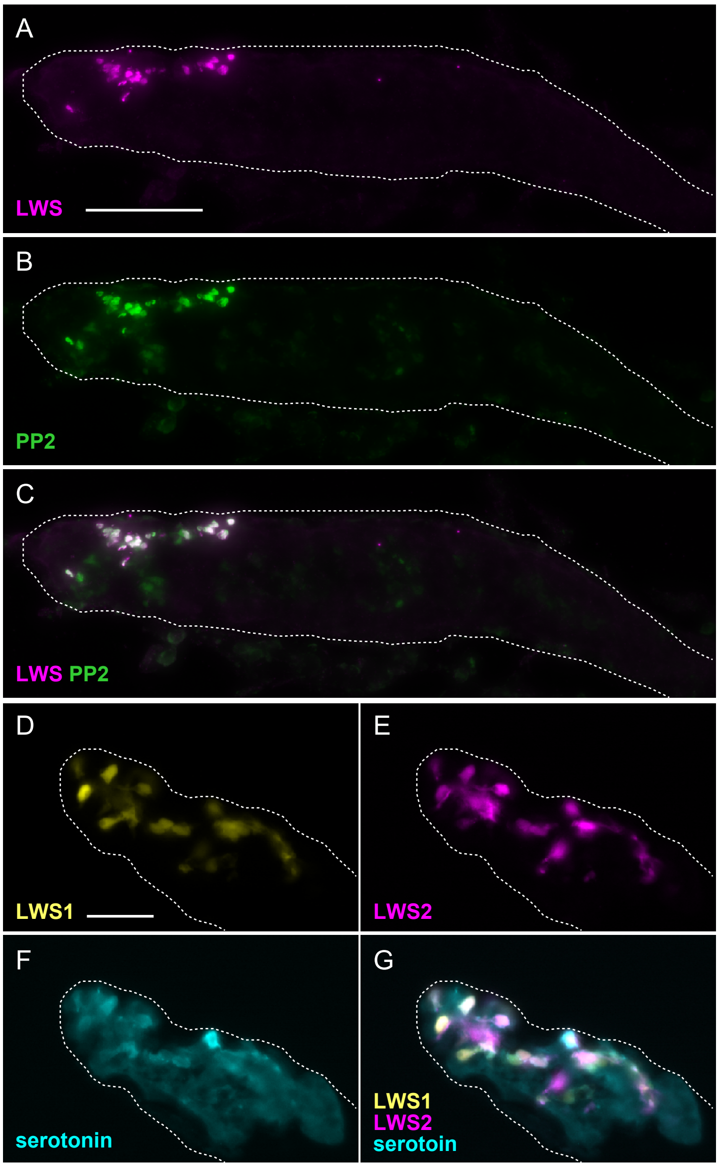

Figure S9. Characterization of LWS opsin-expressing cells in the zebrafish pineal organ. Immunofluorescent labeling of LWS (LWS1 and LWS2) opsin (A) and PP2 (B) in the cells located in the rostral area of the sagittal section of zebrafish pineal organ. (C) Merged image of (A) and (B), showing the expression of LWS opsin in most of PP2-expressing cells in the zebrafish pineal organ. Comparison of LWS1 (D) and LWS2 (E) opsin-expressing cells, which are labeled with GFP and RFP, respectively [63], with serotonin-containing cells (F). (G) Merged image of (D-F), showing that LWS opsin-expressing cells in the zebrafish pineal organ contain serotonin. Note that zebrafish has two LWS genes, LWS1 and LWS2, which are more than 90% identical in their amino acid sequences and have similar absorption spectra [58]. The white dotted traces indicate the landmarks of the pineal organ. The scale bars represent 50 µm.
