# Supplementary material for: Diversification of non-visual photopigment parapinopsin in spectral sensitivity for diverse pineal functions
Source: BMC Biol. 2015 Sep 15;13:73. doi: 10.1186/s12915-015-0174-9 (PMC4570685; doi:10.1186/s12915-015-0174-9)
Supplement: Additional file 10: Figure S10. — Spectroscopic analyses of teleost parapinopsin mutants. (PDF 147 kb) [file 12915_2015_174_MOESM10_ESM.pdf]

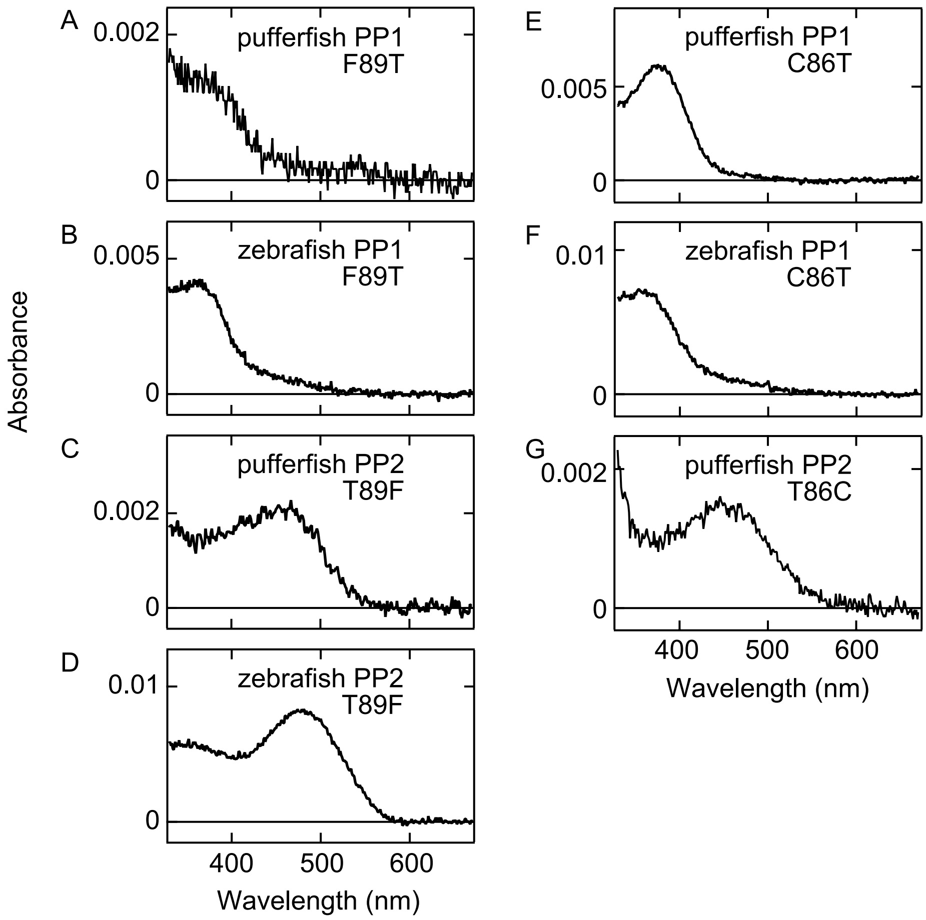

Figure S10. Spectroscopic analyses of teleost parapinopsin mutants. Absorption spectra of the F89T mutants of pufferfish PP1 (A) and zebrafish PP1 (B), T89F mutants of pufferfish PP2 (C) and zebrafish PP2 (D), C86T mutants of pufferfish PP1 (E) and zebrafish PP1 (F), and the T86C mutant of pufferfish PP2 (G) showed no significant spectral changes compared to their respective wild types. Note that the T86C mutant of the zebrafish PP2 was not successfully expressed in cultured cells.
