# Supplementary material for: Diversification of non-visual photopigment parapinopsin in spectral sensitivity for diverse pineal functions
Source: BMC Biol. 2015 Sep 15;13:73. doi: 10.1186/s12915-015-0174-9 (PMC4570685; doi:10.1186/s12915-015-0174-9)
Supplement: Additional file 11: — Accession numbers of amino acid sequences used in the molecular phylogeny inference. (PDF 30 kb) [file 12915_2015_174_MOESM11_ESM.pdf]

Additional File 11

Accession numbers of amino acid sequences used in the molecular phylogeny inference. The sequences were obtained from the NCBI Genbank/RefSeq database (http://www.ncbi.nlm.nih.gov/), Ensembl version 79 (http://www.ensembl.org/), the elephant shark genome project web page (http://esharkgenome.imcb.a-star.edu.sg/), and the coelacanth genome project (LatCha_J1.0) web page (http://coelacanth.nig.ac.jp/).

Figure 1: zebrafish parietopsin, AB693171; lizard parietopsin, DQ100320; lamprey parapinopsin, AB116380; clawed frog parapinopsin, AB159672; iguana parapinopsin, AB626969; spotted gar parapinopsin, ENSLOCP00000017452; pufferfish PP1, AB626964; rainbow trout PP1, AB159673; catfish parapinopsin, AF028014; northern pike PP1, XP_010865161.1; stickleback PP1, ENSGACP00000000245; zebrafish PP1, AB626966; cavefish PP1, ENSAMXP00000015377; pufferfish PP2, AB626965; rainbow trout PP2, AB675727; northern pike PP2, XP_010864048.1; stickleback PP2, ENSGACP00000002101; zebrafish PP2, AB626967; cavefish PP2, ENSAMXP00000007364; marine lamprey P-opsin, U90671; zebrafish VAL, AB035277; toad pinopsin, AF200433; chicken pinopsin, U15762; zebrafish red, NM_131175; human red, AH005298; human green, AH005296; zebrafish UV,

NM_131319; human blue, AH003620; zebrafish blue, NM_131192; chicken blue, M92037; zebrafish green, NP_571329; chicken green, M88178; zebrafish rhodopsin, NM_131084; human rhodopsin, U49742.

Figure S2B: pufferfish CACNA2D3, ENSTRUP00000021957; green pufferfish CACNA2D3, ENSTNIP00000018063; tilapia CACNA2D3, ENSONIP00000015606; human CACNA2D3, NP_060868.2; anole CACNA2D3, ENSACAP00000011746; tilapia cacna2d3, ENSONIP00000009090; green pufferfish cacna2d3, ENSTNIP00000017709; pufferfish cacna2d3, ENSTRUP00000031826; cavefish cacna2d3, ENSAMXP00000007350; cavefish CACNA2D3, ENSAMXP00000015374; anole CACNA2D4, ENSACAP00000010830; clawed frog CACNA2D4, ENSXETP00000001960; human CACNA2D4, NP_758952.4; pufferfish cacna2d4a, ENSTRUP00000041972; green pufferfish cacna2d4a, ENSTNIP00000010390; tilapia cacna2d4a, ENSONIP00000014714; cavefish cacna2d4b, ENSAMXP00000002029; pufferfish cacna2d4b, ENSTRUP00000042388; tilapia cacna2d4b, ENSONIP00000013578; green pufferfish cacna2d4b, ENSTNIP00000000465.

Figure S2C: pufferfish tktb, ENSTRUP00000024713; tilapia tktb,

ENSONIP00000015586; cavefish tktb, ENSAMXP00000013559; clawed frog tktl2, ENSXETP00000034167; anole TKTL, ENSACAP00000012542; chicken TKT, ENSGALP00000008498; anole TKT, ENSACAP00000010793; human TKT, NP_001055.1; clawed frog TKT, ENSXETP00000034164; tilapia TKT, ENSONIP00000002380; green pufferfish TKT, ENSTNIP00000014980; pufferfish TKT, ENSTRUP00000045467; cavefish TKT, ENSAMXP00000015418; tilapia tkta, ENSONIP00000009076; pufferfish tkta, ENSTRUP00000033839; green pufferfish tkta, ENSTNIP00000017713; human TKTL2, NP_115512.3; cavefish tkta, ENSAMXP00000007393.

Figure S2D: pufferfish ERC2, ENSTRUP00000020512; green pufferfish ERC2, ENSTNIP00000002497; tilapia ERC2, ENSONIP00000015625; chicken ERC2, ENSGALP00000008691; human ERC2, NP_056391.1; anole ERC2, ENSACAP00000005842; clawed frog ERC2, ENSXETP00000002025; green pufferfish erc2, ENSTNIP00000017721; pufferfish erc2, ENSTRUP00000034947; tilapia erc2, ENSONIP00000018211; cavefish erc2, ENSAMXP00000014271; tilapia erc1a, ENSONIP00000018664; pufferfish erc1a, ENSTRUP00000031214; green pufferfish erc1a, ENSTNIP00000015077; cavefish erc1a, ENSAMXP00000001903; chicken

ERC1, ENSGALP00000021182; human ERC1, NP_001288177.1; anole ERC1, ENSACAP00000000023; clawed frog ERC1, ENSXETP00000020746; tilapia erc1b, ENSONIP00000014759; pufferfish erc1b, ENSTRUP00000000066; green pufferfish erc1b, ENSTNIP00000008416; cavefish ERC2, ENSAMXP00000015353.
